# Protonation of histidine rings using quantum-mechanical methods

**DOI:** 10.1107/S2059798324006314

**Published:** 2024-07-25

**Authors:** Nigel W. Moriarty, Jonathan Moussa, Paul D. Adams

**Affiliations:** a Molecular Biophysics and Integrated Bioimaging Lawrence Berkeley National Laboratory Berkeley CA94720 USA; b Molecular Sciences Software Institute Virginia Tech Blacksburg VA24060 USA; c Department of Bioengineering University of California Berkeley Berkeley CA94720 USA; F. Hoffmann-La Roche Ltd, Switzerland

**Keywords:** macromolecular refinement, histidine, quantum-mechanical predictions

## Abstract

Histidine can be protonated on either or both of the two N atoms of the imidazole moiety. Each of the three possible forms occurs as a result of the stereochemical environment of the histidine side chain. In an atomic model, comparing the possible protonation states *in situ*, looking at possible hydrogen bonding and metal coordination, it is possible to predict which is most likely to be correct. A more direct method is described that uses quantum-mechanical methods to calculate, also *in situ*, the minimum geometry and energy for comparison, and therefore to more accurately identify the most likely proton­ation state.

## Abstract 2

Histidine protonation is fraught, but a new quantum-mechanical method can provide insight into choices.

## 1. Introduction

Protonation of histidine can take on three different forms: one H atom on either or both of the N atoms in the heterogeneous ring of the imidazole moiety. As noted by Malinska *et al.* (2015 ▸)

This variability is of particular importance in protein structures, although it is also particularly recalcitrant to X-ray crystallo­graphic characterization because of the limited resolution and the inability to detect H atoms that blight the method in routine applications.

This obstinate behaviour led to a procedure to determine the protonation of the histidine by matching the refined ring geometry with those in the Cambridge Structural Database (CSD; Groom *et al.*, 2016 ▸). The protonation-determination procedure involved relaxing the restraints of the histidine side chain and using discrimination functions based on the CSD data to predict the protonation. The example used was PDB entry 4rj2: a 0.99 Å resolution model of *Escherichia coli* purine nucleoside phosphorylase that comprises a hexamer of sub­units (*A*–*F*), each containing six histidine residues. An upper *B*-factor limit of 20 Å^2^ on the post-refinement N atom was used to filter for accuracy.

The procedure was used to predict the presence or absence of a particular proton, resulting in some ambiguity in the final protonation. Unfortunately, histidine remained unruly as the method was only able to predict 61% of the 144 (completely ordered) proton positions in the model. It does, however, provide a valuable curated test set of histidine protonation.

Recently, the power of quantum-mechanical (QM) calculations was applied to the generation of restraints using the *in situ* geometries of the target molecule (Liebschner *et al.*, 2023 ▸). Known as *QM Restraints* (*QMR*), this program is part of the *Quantum Interface* (*QI*) in the *Phenix* suite of programs (Liebschner *et al.*, 2019 ▸). *QI* is a central location for interacting with quantum-mechanical modules in *Phenix*. The power of QM is that it does not require atomic properties, such as van der Waals radii, to be known in order to calculate the protonation state. QM methods rely on the specification of element and electron positions alone, minimizing the electron wavefunction to determine properties of the molecule. This makes them orthogonal to and independent of the methods used for many of the related macromolecular computational methods.

A key feature of the *QI* module is the ability to create a selection of the overall model representing the molecular environment of the entity of interest. The default mechanism is to include all entities such as amino acids, metals, waters and ligands within a buffer distance (with a default of 3.5 Å) of the user-defined selection. For a drug candidate, this selection will likely be a small molecule in a protein binding pocket. *QI* also prepares this small model (or cluster) for QM calculations by adding H atoms to solvent and appropriately protonating the protein main-chain terminations created by the selection process. The surrounding amino acids (and other entities) are frozen while the geometry of the ligand and all H atoms are minimized. By default, all of the atoms in the user-defined selection and the H atoms in the remainder of the cluster are energy-minimized using the QM method of choice. The minimized geometry is used to create geometric restraints that reflect the molecular environment of the binding pocket, which are then used in standard crystallographic refinement algorithms (Afonine *et al.*, 2012 ▸). Two QM packages are currently supported: *MOPAC* (Stewart, 1990 ▸; Moussa & Stewart, 2024 ▸), which is distributed with the Python3 version of *Phenix*, and *Orca* (Neese *et al.*, 2020 ▸), which is a third-party QM package supported by the *QI* module. This integration of *MOPAC* and *Phenix* means that nuances in the energy calculations used in this work can be handled automatically by the code (see the discussion below). *Orca* is also well suited to the *QMR* module, particularly for ligands with novel moieties. The default QM method when using *MOPAC* is PM6-D3H4, while the default method for *Orca* is PBEh-3c; however, this is easily changed.

For *QMR*, the entity of interest is usually a drug molecule or other ligand. However, any reasonable selection can be used to create a *QI* model, including, for example, a nonstandard amino acid, should the restraints for the side chain be needed. For the standard amino acid histidine, the protonation of the imidazole moiety is affected by the molecular environment which is extant in the selected QM model.

## 2. Methods

### 2.1. Histidine configurations

As stated earlier, there are three protonation states: an H atom on either or both of the N atoms in the heterogeneous ring. Each configuration can be identified by the name of the proton: HD1 and HE2 for the singly protonated states and, using the logical ‘and’, HD1^HE2 for the double protonation. It should be noted that the HE2 tautomer is slightly more stable than the HD1 tautomer. In addition, histidine side-chain rings can rotate around the χ_2_ torsion, generating the possibility of two conformations 180° apart. This is part of the NQH flipping algorithm implemented in the *MolProbity* suite (Williams *et al.*, 2018 ▸) as part of the hydrogen-adding program *reduce*. In general, the orientations of the N atoms in the ring are correct in a refined model. However, to cover all possibilities, including the rarer rotamers, possible flips of the side chain add three more possible conformations.

**Figure 1:**
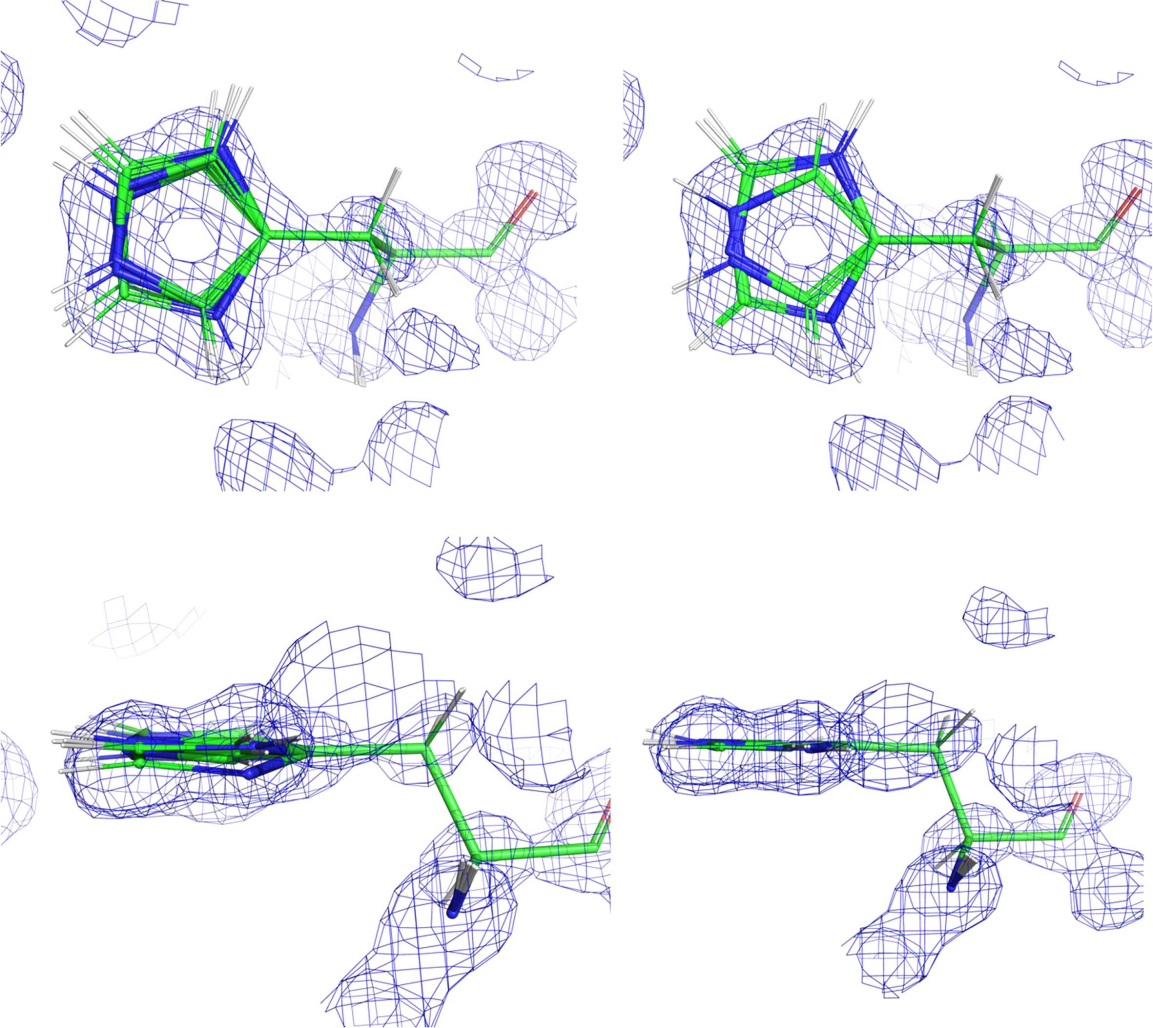
Comparison of geometry minimizations of the six starting configurations of histidine residue 4 in chain *A* of PDB entry 4rj2. The left column allows the planar torsion to relax, whilst the right column maintains the starting position. The top views are perpendicular to the ring, with the bottom views showing the ring on edge. Note that the atoms from the main-chain CA atom to the CG atom are frozen in both cases.

A new program, *QM Flipping* (*QMF*), was developed to determine which of these six starting protonations/flips is most likely in an atomic model. Each of the six configurations are geometry-minimized to ascertain their feasibility. A geometry minimization can change the atomic positions greatly. As in the *QMR* calculations, the protein-environment atomic positions are frozen except for the H atoms. Because the histidine is the entity of interest, its atoms have more freedom. Allowing complete freedom produces spurious geometries, particularly in cases where the proposed protonation causes a clash. However, because the position of the histidine is reasonably well known, one can leverage the experimental information to determine the plane of the ring. Thus, the default has the plane of the ring restricted via the torsion angle towards the main chain. This results in better geometries for ‘bad’ protonations, as can be seen by comparing the left side of Fig. 1 ▸ (unrestricted) with the right (restricted). In this particular high-resolution example, because the water molecules have been placed the changes in geometries are minimal, but are still sufficient to extend outside the density. However, in poorly defined environments the final geometries can be very distorted.

### 2.2. Heats-of-formation comparisons

One additional attribute of QM-minimized geometries is the calculated minimum energy. These can be compared directly if the same number of atoms and electrons are in each configuration. For the trivial cases of asparagine and glutamine (‘NQ’ of ‘NQH flips’) this is the case, but for histidine two of the six configurations have an additional proton. Parenthetically, the planar restriction also results in higher energies for ‘bad’ protonations, providing more discrimination between states. An additional note for comparisons of the NQH flips is that each cluster needs to contain the same number of entities. This nuance required that the cluster needed to be chosen via a selection syntax rather than the buffer parameter, a subtlety automatically handled via the *QI* interface for *QMF*.

The semi-empirical QM models implemented in *MOPAC* are primarily intended for thermochemistry applications defining a heat of formation for each molecular configuration. These heats are relative to the standard state of the constituent atoms and a reservoir of electrons in vacuum. The relative stability of two configurations can be directly predicted by the difference between their heats of formation when they contain the same number of atoms and electrons. Otherwise, the difference implies a reaction transferring atoms and electrons to or from their standard reference states. In the case of an excess proton, the reference state is derived from molecular hydrogen gas, which includes neutralizing electrons taken from vacuum. This is an unphysiological reaction for biological applications, where the reference state of a proton is assumed to be solvation in water with a pH of 7.4. To account for this change of reference, the free energies reported by *MOPAC* need a correction term for each excess or deficiency of a proton to compare them with a neutral molecular configuration for biological applications. In some cases, heats of formation are also modified by an implicit solvent model approximating the dielectric response of the chemical environment that is not explicitly contained in the calculation.

To assign a biologically relevant proton heat of formation, we follow an established approach of fitting QM data to a simple semiempirical model for p*K*_a_ (Matsui *et al.*, 2012 ▸),
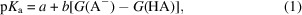
where *a* and *b* are model parameters, *G*(HA) is the calculated heat of formation of solute molecule A and *G*(A^−^) is the calculated heat of formation of the deprotonated solute. The model parameters will depend on the specific semiempirical model, and here we fit the correction for the PM6-D3H4 model (Řezáč & Hobza, 2012 ▸). In Table 1 ▸, we fit this model to minimize the root-mean-square (r.m.s.) error for 20 experimental reference values of p*K*_a_ that include both N and O atoms as protonation sites. In these data, the amino acids arginine, histidine, lysine, aspartic acid, glutamic acid and tyrosine are in the zwitterionic form and are protonated on their side chains. These PM6-D3H4 calculations use COSMO implicit solvent with a relative static permittivity of 78.4 to approximate the aqueous environment (Klamt & Schüürmann, 1993 ▸). The minimum r.m.s. error of 1.77 is achieved with *a* = 42.18 and *b* = 0.368 mol kcal^−1^. The effective free-energy correction for the proton balances the heats of formation of protonated and unprotonated solutes when the p*K*_a_ matches the pH,
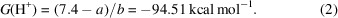


More accurate, but less transferable, p*K*_a_ and *G*(H^+^) values can be obtained by fitting models for specific protonation sites. For example, a fit to only nitrogen-bound protons results in *G*(H^+^) = −94.72 kcal mol^−1^, while a fit to only oxygen-bound protons results in *G*(H^+^) = −93.88 kcal mol^−1^. We use the general-purpose value of −94.51 kcal mol^−1^ since it is close to the special-purpose value for nitrogen-bound protons such as in histidine.

The accuracy of the QM fitting described here provides an r.m.s. error for comparing heats of formation when the additional proton is involved. The r.m.s. error of the p*K*_a_ is 1.77, which translates to an r.m.s. error of 4.81 kcal mol^−1^ when translated to an energy using our semiempirical linear model. The model cannot reliably order states with an energy difference less than this value. The errors in this p*K*_a_ model come from errors in the semiempirical QM model, errors in approximating the chemical environment by an implicit solvent model and errors from using a single, minimum-energy geometry for solute molecules in implicit solvent. The largest error in Table 1 ▸ is for arginine, which is likely to come from the PM6-D3H4 model because the guanidino group in its minimum-energy geometry of arginine has poor planarity, and geometric errors typically imply energetic errors.

While the p*K*_a_ model used here can be paired with other QM-based model chemistries, it may need to be refitted for different levels of theory and adjusted for different energy references and applications. For example, previous fits of this model (Matsui *et al.*, 2012 ▸) used density-functional theory (DFT) calculations, a reference proton free energy of zero and fit different model parameters for protons bound to six different side groups. Adjusting for the change in reference used in *MOPAC*, this fitting produced a range of proton heats of formation between −101.49 and −69.31 kcal mol^−1^, with an average heat of −85.54 kcal mol^−1^.

### 2.3. Other metrics

Additional metrics to assess each histidine configuration can be helpful. Recently, a program for determining the model quality based on hydrogen bonding (Afonine *et al.*, 2023 ▸) was added to *Phenix*. As part of the validation of histidine conformations, hydrogen bonds are counted. When applied to each of the final six histidine configurations, an increase in hydrogen bonds will generally lead to a better result. Another metric is based on the movement of the minimized atoms. A large root-mean-square deviation (r.m.s.d.) of the starting and final geometries could be due to a poor protonation clashing with the surrounding amino acids. One final metric is the rotameric state (Hintze *et al.*, 2016 ▸; Williams *et al.*, 2018 ▸). This rarely changes except between the two flip states, but is reported for information. If the state is labelled ‘OUTLIER’ then it is a case for further investigation.

## 3. Results

### 3.1. Single-histidine example

The output of a single *QMF* run is summarized in Table 2 ▸. The original configuration is displayed on the first line with its rotamer. Each subsequent line lists the configuration, energy, Δ*E*, number of hydrogen bonds, r.m.s.d. from the starting geometry and rotamer. The first three calculated geometries differ only in the number and position of protons (see Fig. 1 ▸). This is reflected in the rotamer (all of which are the same) and the r.m.s.d. values. The larger r.m.s.d. values for the flipped configurations are due to the swapping of the Cartesian coordinates to flip the ring by 180°.

**Figure 2:**
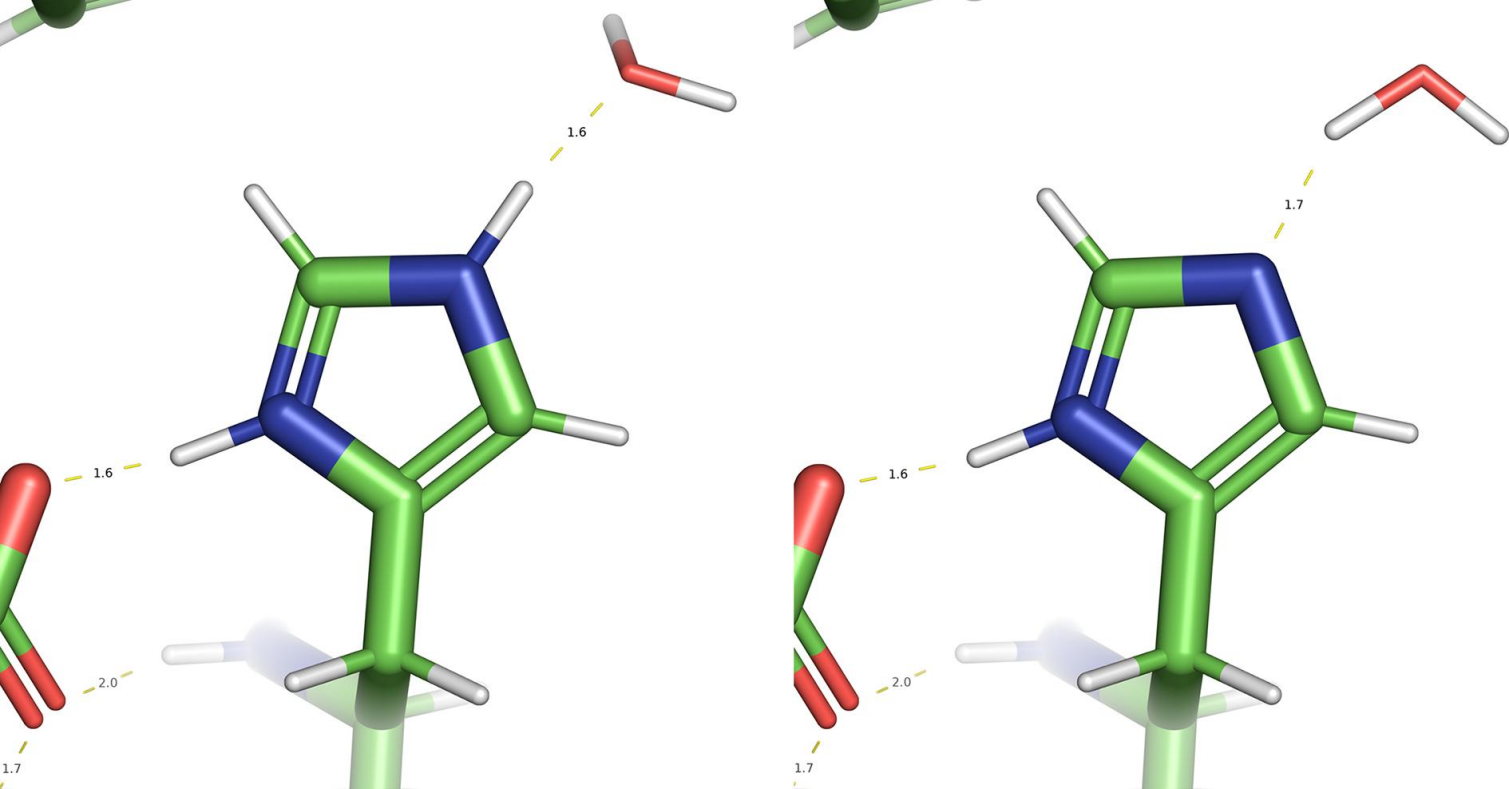
Minimized geometry of two histidine configurations of histidine in chain *A* and residue 4 of PDB entry 4rj2.

The number of hydrogen bonds is greatest for the doubly protonated and HD1 protonation states. At 14, it is one greater than that for HE1, which is reflected in the Δ*E* of 19.3 kcal mol^−1^ between the HD1 and HE2 configurations. The energy difference between doubly protonated and HD1 protonation is 2.6 kcal mol^−1^, which is well below the significance level (4.81 kcal mol^−1^). Closer inspection of the two geometries (Fig. 2 ▸) shows that the nearby water molecule is involved with the HE2 proton in the doubly protonation configuration and with the NE2 N atom in the HD1 state, thus maintaining the same number of hydrogen bonds.

### 3.2. Comprehensive protein environment example

Extending the use of *QMF* to all histidine amino acids in the high-resolution example PDB entry 4rj2 is displayed in Table 3 ▸. The first row of the table displays the results discussed in the example in Table 2 ▸ and Fig. 2 ▸. The results from Malinska and coworkers are shown in the second and third columns. A ‘+’ means protonated, a ‘−’ means unprotonated and a ‘?’ means unknown. For the example of the histidine labelled as residue 4 in chain *A* (compactly ‘His 4 A’), it shows that ND1 is protonated, but it is unclear about the protonation state of NE2. Both *MolProbity* and *QMF* agree with the addition of HD1, but differ on the presence of HE2. As already mentioned, *QMF* calculates an insignificant Δ*E*, indicating that either double protonation (HD1^HE2) or HD1 proton­ation is acceptable.

The next histidine of interest is His 97 A. Malinska and coworkers do not have a prediction due to high *B*-factor values that are a result of the amino acid being on the surface of the protein. Consequently, there are few hydrogen bonds involved, thus lowering the discriminating power of *QMF*. A closer look at the density around this histidine shows the possibility of one or two water molecules. If these solvent molecules could be justified, they may provide more discrimination. The same situation is true for His 209 A, a surface side chain with possible water interactions. The relevance of the protonation of a surface histidine is low, so the poor discrimination of the method is less important. Removing these special cases from all chains results in perfect agreement between the methods; in particular, *MolProbity* and *QMF* agree in all cases.

### 3.3. Metal-coordination example

**Figure 3:**
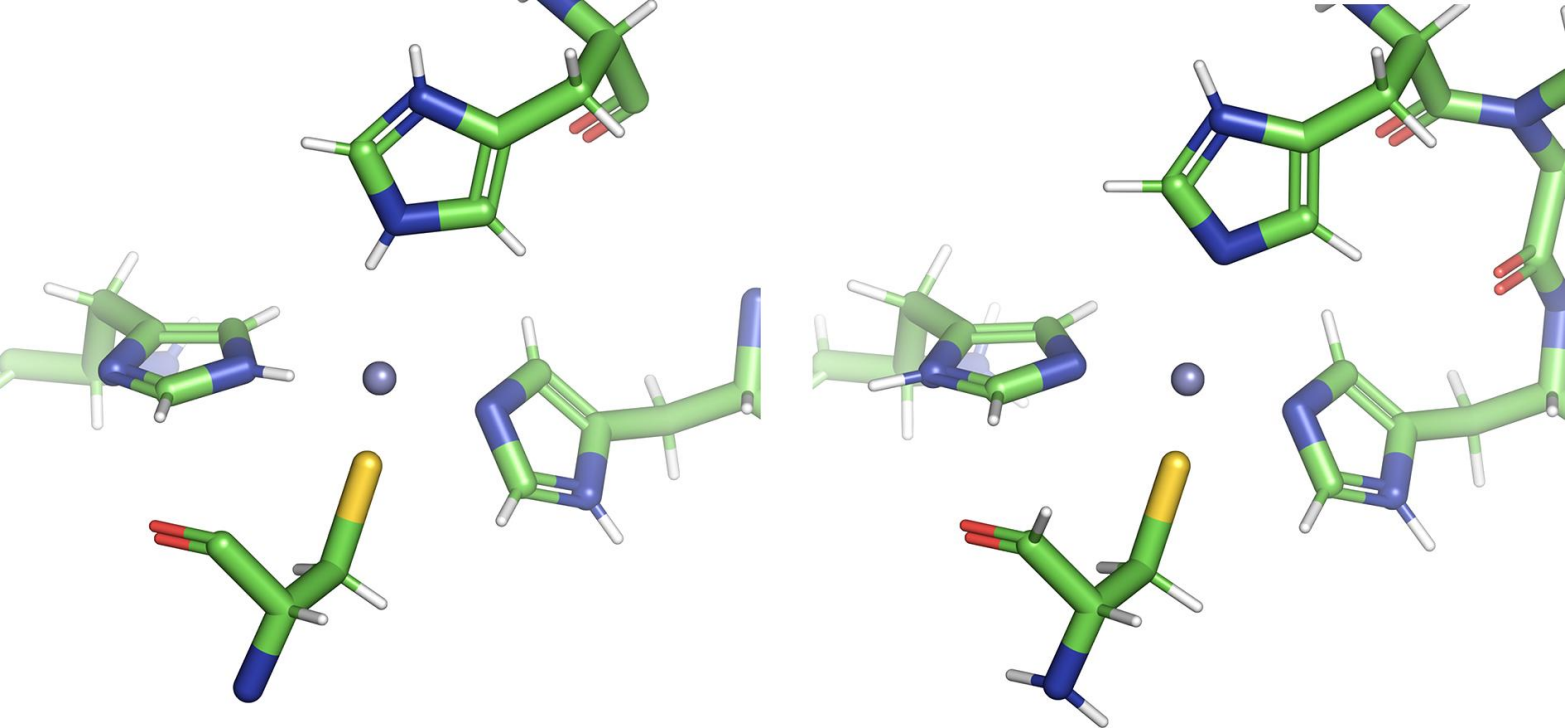
Metal-coordinated protonation states of the three histidine side chains around Zn 4 B in PDB entry 3rzu as predicted by *MolProbity* (left) and *QMF* (right).

Histidine coordinates with many metal ions, with the zinc finger being a common motif. The correct protonation of a metal ion is particularly important, as an incorrectly placed proton can distort the final refined model. This would appear to be the case for Zn 4 B in PDB entry 3rzu, as shown in Fig. 3 ▸. The doubly protonated histidine at the top of the image is 2.63 Å from the zinc, much farther than the more accurate 2.1–2.3 Å range. The other histidines are also distorted.

*MolProbity* protonates the model as shown in the left panel; however, *QMF* predicts the correct protonation depicted on the right. Furthermore, the smallest energy difference between the lowest energy state and the next lowest state was 21 kcal mol^−1^, with the majority being far larger. In cases where the proton is pointing towards the metal, it was often bent out of the plane of the imidazole ring. Naturally, this spurious geometry was reflected by a high energy.

It is noteworthy that the ‘wrong’ states of the other histidines coordinated with the zinc had little impact on the final energy differences, but an approach that corrects each histidine before proceeding to the next hisitidine and revisiting each to ensure a consistent result may be advisable.

### 3.4. Ligand-environment example

**Figure 4:**
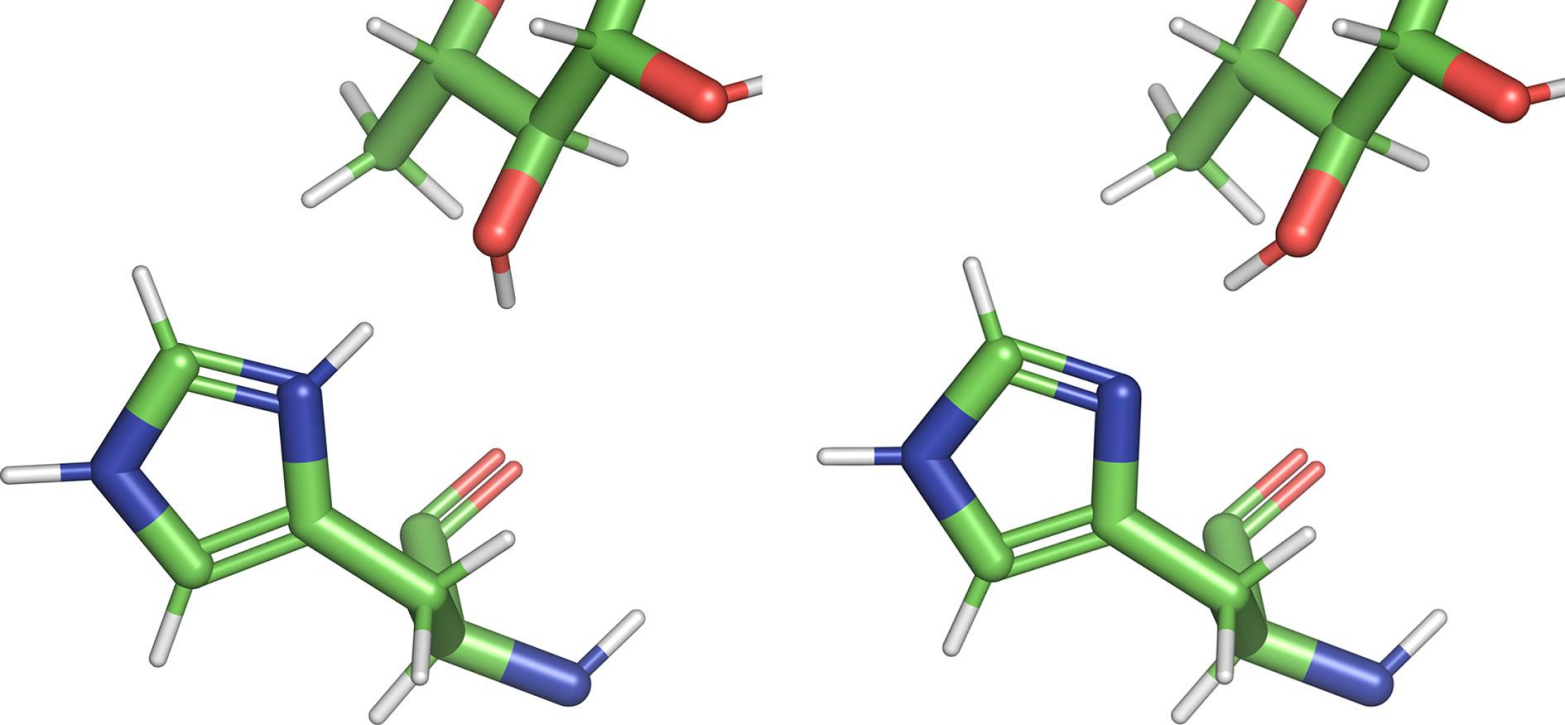
Two lowest energy configurations of His 119 B from PDB entry 2ixc. The right panel (HE2 protonated) is 20.4 kcal mol^−1^ lower in energy than the left panel (doubly protonated).

An example of a histidine interacting with a ligand can be found in PDB entry 2ixc, a carbohydrate epimerase resolved to 1.8 Å. The ligand (2′-deoxythymidine-β-l-rhamnose, code TRH) has a carbohydrate-like structure at one end that interacts with two histidine side chains. All four copies of TRH pass the polder OMIT map (Liebschner *et al.*, 2017 ▸) test, so the chain *B* copy was chosen randomly. His 119 B has a similar motif to the single-histidine example in that one proton in a doubly protonated configuration is a hydrogen-bond donor, but when the proton is removed the N atom becomes an acceptor (Fig. 4 ▸). In the previous example, the interaction is with a water molecule, while in this example it is the carbohydrate moiety.

In contrast, the energy difference favours HE2 protonation by 20.4 kcal mol^−1^, which is far more significant than the value of 2.6 kcal mol^−1^ in the first example. Similarly, *MolProbity* predicts a doubly protonated state in this example. The resolution precludes using the discrimination functions method. The difference in vapour energies (David *et al.*, 2017 ▸) favours the N-atom acceptor (O—H⋯:N) by 5 kcal mol^−1^ over the O-atom acceptor (N−H⋯:O). Other factors affect the final heat of formation, but in this case the increased strength of the hydrogen bond appears to be adding to the improvement in energy for the singly protonated configuration. It should be noted that the protonation state of His 62 B agrees with *MolProbity* and is not changed for this comparison.

**Figure 5:**
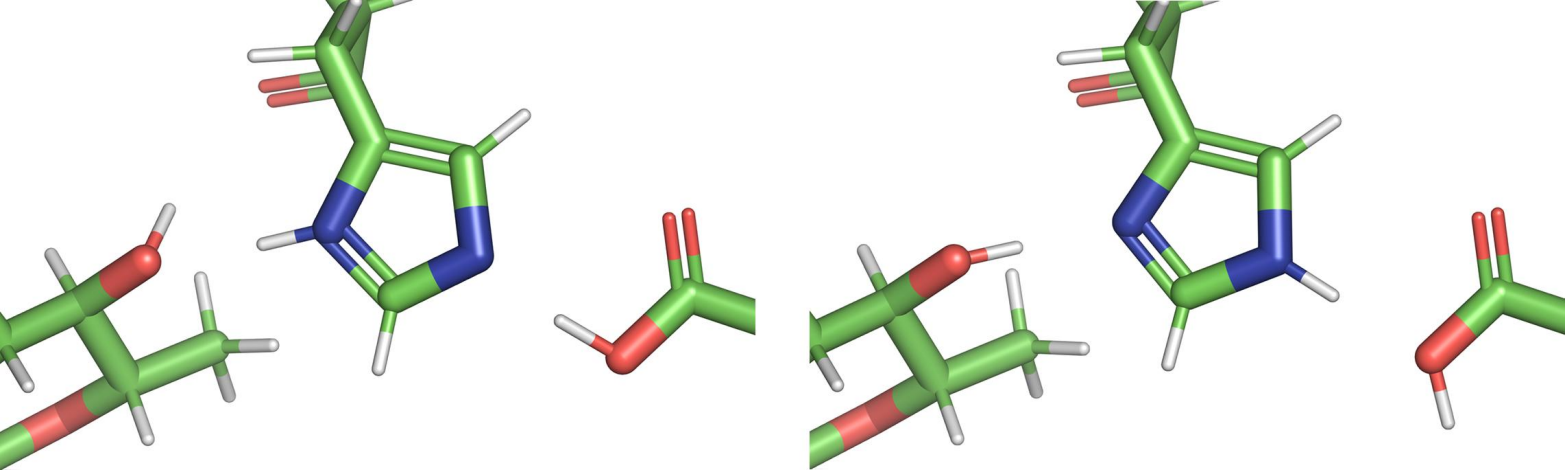
Two lowest energy configurations of His 119 B from PDB entry 2ixc. The right panel (HE2 protonated) is 22.8 kcal mol^−1^ lower in energy than the left panel (HD1 protonated).

Other amino-acid side chains in the vicinity can affect the local pH. In this particular case, protonation of Asp 83 B could affect the protonation state of His 119 B. To test this hypo­thesis, Asp 83 B was protonated and the model was refined. The *QMF* procedure was then repeated. Once again, the HE2 protonated state was lowest in energy: by 22.8 kcal mol^−1^ compared with the HD1 protonated state. These two states are shown in Fig. 5 ▸. The lowest energy state has the same hydrogen bond (His 119 B is the acceptor) as the unprotonated Asp 83 B model. The position of the proton of Asp 83 B rotates away for the HE2 protonation and rotates towards His 119 B in the HD1 state. The doubly protonated state is 42.3 kcal mol^−1^ higher in energy than the HE2 protonated state.

## 4. Conclusions

Accurate prediction of histidine protonation is not a trivial task and X-ray crystallographic data do not provide direct information about protonation, except with very high-resolution data. Acceptable resolution neutron data can be a path to more information. The *MolProbity* suite provides an empirical approach that optimizes the hydrogen-bonding network. This is one of its strengths and should be relied on to perform this function for large networks in standard proteins. Malinska and coworkers proposed geometry-based discrimination functions that are limited to high-resolution structures with low temperature factors. Here, we show that *Quantum Mechanical Flipping* (*QMF*) using QM energies and hydrogen-bonding metrics can predict histidine protonation in agreement with both *MolProbity* and Malinska and coworkers in the high-resolution example. *QMF* will also work at low resolution due its reliance solely on the atomic positions, meaning that any well characterized region of the model is viable.

Overall, the *QMF* method has the advantage of working at typical resolutions in the macromolecular structure determination field as well as being capable of determining histidine protonations in environments including nonstandard amino acids, metal ions and ligands, including drug-like entities, where *MolProbity* may fail due to its limited parameterization. *QMF* is also crystal symmetry-aware, so interactions between symmetry copies are addressed by the method.

*QMF* does not use the structure factors. Nor does it validate the position of any atoms in the experimental data: this is the user’s responsibility. It can therefore be used with either X-ray or cryoEM models that are reasonable in the region of interest. As shown in the ligand example, various protonation states in the region of interest can be investigated.

One case is not covered explicitly in this article: a non­standard amino acid with an imidazole or similar moiety (for example d-histidine, DHI) or a ligand with a titratable group. A user can use these tools to calculate the energy and other metrics with a little care. Neither of the previous methods can help; however, *QI* has the tools available for the user to manually minimize the geometry of each important state and calculate the energies. Each state needs to be generated by the user and passed to *QMR*. A simple energy comparison (including the protonation energy adjustment as appropriate) as well as looking at each geometry can provide the necessary answers.

## 5. Availability

The *Quantum Mechanical Restraints* (*QMR*) and *Quantum Mechanical Flipping* (*QMF*) modules of the *Quantum Interface* (*QI*) are available in version 1.21.1-5286 of the *Phenix* package. Downloading the Python3 version will include *MOPAC*, which can be installed separately for the official Python2 version. Downloading a nightly build will provide more features for both *QMF* and the *QI* modules in general.

## Figures and Tables

**Table 1:** Experimental and model p*K*_a_ values Experimental data were sourced from Wikipedia, either the entry for the molecule, the entry for ‘amino acid’ or the entry for ‘acid dissociation constant’.

Solute molecule	Experimental p*K*_a_	Model p*K*_a_	*G*(A^−^) − *G*(HA) (kcal mol^−1^)
Arginine	13.8	8.57	−91.32
Histidine	6.0	6.19	−97.80
Lysine	10.7	10.71	−85.52
Ammonia	9.25	11.04	−84.62
Methylamine	10.65	10.77	−85.35
Pyridine	5.2	6.39	−97.26
Aniline	4.6	6.53	−96.88
Pyrrolidine	11.4	9.89	−87.75
Adenine	4.17	4.54	−102.29
Adenine (deprotonated)	9.65	9.00	−90.17
Aspartic acid	3.90	3.21	−105.89
Glutamic acid	4.07	5.11	−100.74
Tyrosine	10.1	8.08	−92.67
Water	14.0	14.19	−76.06
Hydrogen peroxide	11.75	13.75	−77.26
Formic acid	3.75	5.91	−98.57
Phenol	9.95	8.41	−91.76
Benzoic acid	4.20	5.16	−100.60
Oxalic acid	1.27	0.85	−112.30
Oxalic acid (deprotonated)	4.27	4.54	−102.29

**Table 2:** The *QMF* results for histidine residue 4 in chain *A* of PDB entry 4rj2

	Configuration	Energy (kcal mol^−1^)	Δ*E* (kcal mol^−1^)	Hydrogen bonds	R.m.s.d. (Å)	Rotamer
0	HD1, HE2					**m**90
1	HD1, HE2	−1019.6	2.6	14	0.04	**m**90
2	HD1 only	−1022.2	0.0	14	0.04	**m**90
3	HE2 only	−1003.0	19.3	13	0.05	**m**90
4	HD1, HE2 flipped	−1004.0	18.3	12	0.29	**m**-70
5	HD1 only flipped	−1004.4	17.9	12	0.38	**m**-70
6	HE2 only flipped	−1009.4	12.9	11	0.32	**m**-70

**Table 3:** Summary of predicted histidine protonation states for PDB entry 4rj2 For the results from Malinska and coworkers, each N-atom proton is labelled ‘+’ for present, ‘−’ for absent and ‘?’ for unknown. In the comparison columns, ‘Y’ and ‘N’ are yes and no, respectively, while ‘M’ means maybe. The results from *MolProbity* and *QMF* are included as ‘HD1^HE2’ for doubly protonated, with a single atom name for the singly protonated results.

	Malinska and coworkers						
	HD1	HE2	*MolProbity* versus Malinska	*QMF* versus Malinska	*MolProbity*	*QMF* versus *MolProbity*	*QMF*
His 4 A	+	?	M	M	HD1^HE2	N	HD1
His 62 A	+	−	Y	Y	HD1	Y	HD1
His 97 A	?	?	?	?	HD1	N	HE2
His 123 A	−	+	Y	Y	HE2	Y	HE2
His 205 A	−	+	Y	Y	HE2	Y	HE2
His 209 A	?	?	?	?	HE2	Y	HE2
His 4 B	+	?	M	M	HD1	N	HD1^HE2
His 62 B	+	−	Y	Y	HD1	Y	HD1
His 97 B	?	?	?	?	HD1	Y	HD1
His 123 B	−	+	Y	Y	HE2	Y	HE2
His 205 B	?	?	?	?	HE2	Y	HE2
His 209 B	?	?	?	?	HE2	Y	HE2
His 4 C	+	−	N	Y	HD1^HE2	N	HD1
His 62 C	+	−	Y	Y	HD1	Y	HD1
His 97 C	?	?	?	?	HE2	Y	HE2
His 123 C	−	+	Y	Y	HE2	Y	HE2
His 205 C	−	+	Y	Y	HE2	Y	HE2
His 209 C	?	?	?	?	HE2	N	HD1
His 4 D	+	?	M	M	HD1^HE2	N	HD1
His 62 D	+	−	Y	Y	HD1	Y	HD1
His 97 D	?	?	?	?	HE2	Y	HE2
His 123 D	−	+	Y	Y	HE2	Y	HE2
His 205 D	+	+	Y	Y	HD1^HE2	Y	HD1^HE2
His 209 D	+	+	N	N	HD1	Y	HD1
His 4 E	+	?	M	M	HD1	Y	HD1
His 62 E	+	−	Y	Y	HD1	Y	HD1
His 97 E	?	?	?	?	HD1	Y	HD1
His 123 E	−	+	Y	Y	HE2	Y	HE2
His 205 E	−	+	Y	Y	HE2	Y	HE2
His 209 E	?	?	?	?	HE2	Y	HE2
His 4 F	+	?	M	M	HD1^HE2	N	HD1
His 62 F	+	?	M	M	HD1	Y	HD1
His 97 F	−	+	N	Y	HD1	N	HE2
His 123 F	−	+	Y	Y	HE2	Y	HE2
His 205 F	−	+	Y	Y	HE2	Y	HE2
His 209 F	?	?	?	?	HE2	Y	HE2
